# E-health treatments for Dual Disorders on pregnancy

**DOI:** 10.1192/j.eurpsy.2022.272

**Published:** 2022-09-01

**Authors:** R. Carmona Camacho, N. Lopez Carpintero, I. Caro-Cañizares, L. Albarracin García, E. Baca Garcia, M. Sanchez Alonso

**Affiliations:** 1 Hospital Fundación Jiménez Díaz, Psychiatry, Madrid, Spain; 2 Tajo University Hospital, Gynecology And Obstetrics, Aranjuez, Spain; 3 Universidad a Distancia de Madrid (UDIMA), Psychology, Collado Villalba, Spain; 4 Universidad Autonoma MAdrid, Psychiatry, Madrid, Spain

**Keywords:** perinatal mental health, dual disorders, Perinatal care

## Abstract

**Introduction:**

Dual pathology during pregnancy, described as the co-occurrence of substance use and mental health problems, is one of the leading preventable causes of maternal and perinatal mortality and morbidity; however, effective and accessible treatments are lacking.

**Objectives:**

As part of the WOMAP(Woman Mental Health and Addictions on Pregnancy) initiative, our study aimed to evaluate the effectiveness of an e-health-based psychotherapeutic program compared to enhanced usual care.

**Methods:**

This effectiveness clinical trial was conducted between 2016-2020 in 5 hospitals in the Madrid (Spain) metropolitan area. 2014 pregnant women under 26 weeks of pregnancy were screened. Eligible participants(n=120) were those who screened positive for co-occurring symptoms (AC-OK screener) and were not receiving specialized behavioral treatment. Participants were assessed in depth at baseline, 2,4,8 and 12 months(PHQ-9;GAD-7;PCL-5;AUDIT;DAST;Fagerström) and randomized to the usual care control group(n=38) or to two groups of a 10-session pregnancy-adapted psychotherapeutic program, one delivered by App/internet(n=41) and one by telephone(n=41). Intent-to-treat analyses assessed effectiveness.

**Results:**

Statistically significant effects of the intervention were found for mental health symptoms in the telephone group as compared to the control and App/internet groups, with an improvement effect starting earlier (2 months) and lasting longer (figures 1-3). Regarding substance use, due to the lack of other substances consumption, only smoking and alcohol cessation rates were analyzed. Patients in the App/internet and telephone groups discontinued significantly more, earlier and for a longer period compared to the control group(figures 4-5).

**Figure:**
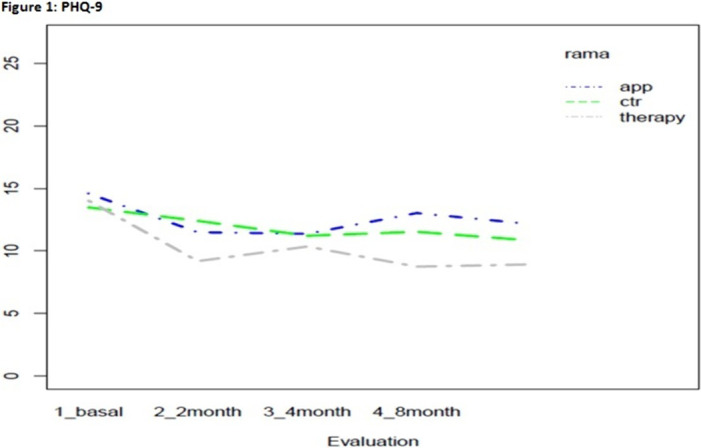

**Figure:**
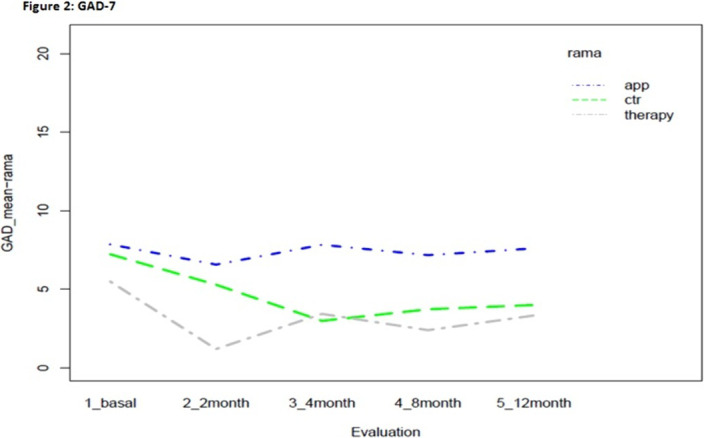

**Figure:**
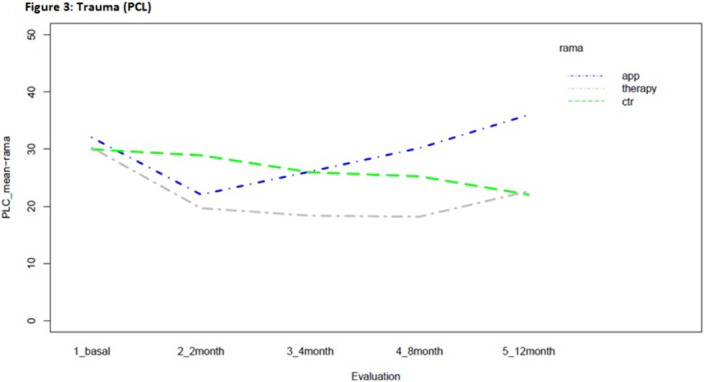

**Figure:**
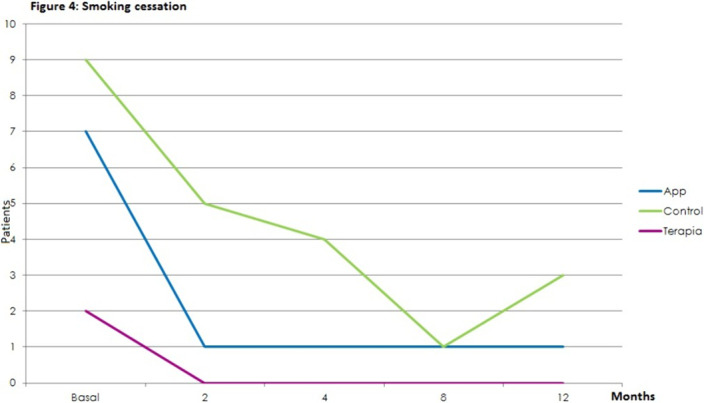

**Figure:**
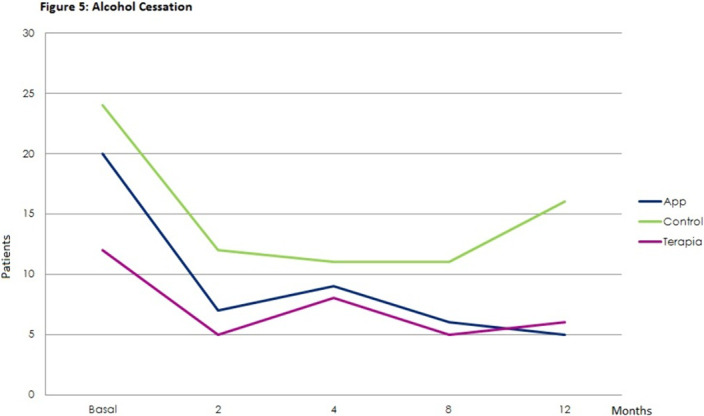

**Conclusions:**

E-health psychotherapeutic programs could benefit pregnant women with dual disorders. An App/internet implementation could only be useful if focused solely on substances.

**Disclosure:**

No significant relationships.

